# Influence of Untreated and Microbially Degraded Mangrove Sediment Microplastics on *Zebrafish* (*Danio rerio*) Intestinal Histology and Immune and Antioxidant Biomarkers

**DOI:** 10.3390/vetsci12090854

**Published:** 2025-09-04

**Authors:** Xin-Yu Zheng, Wan Wei, Asim Muhammad, Min Zhang, Yan-Jun Chen, Jia-Hong Xie, Dan-Ju Kang, Jin-Jun Chen

**Affiliations:** Department of Veterinary Medicine, School of Coastal Agriculture, Guangdong Ocean University, Zhanjiang 524088, China; 18604068009@stu.gdou.edu.cn (X.-Y.Z.); 19806873590@163.com (W.W.); asimmuhammad9862@gmail.com (A.M.); 13634322028@163.com (M.Z.); chenyanjun2@stu.gdou.edu.cn (Y.-J.C.); 18758019771@stu.gdou.edu.cn (J.-H.X.); kangdanju@126.com (D.-J.K.)

**Keywords:** microplastics, degradation, mangrove sediments, oxidative stress, gene expression, immune response, intestinal toxicology, *zebrafish*

## Abstract

MPs are pervasive pollutants in marine ecosystems, posing risks to aquatic organisms due to their small size and bioaccumulation potential. This study investigated the intestinal toxicity of MP particles extracted from mangrove sediments in *zebrafish*, comparing the effects before and after microbial degradation. *Zebrafish* were exposed to either undegraded MPs or microbially degraded MP extracts at concentrations of 0 (control), 2, 10, and 50 mg/L for 21 days in 10 L tanks (stocking density: 10 fish/L), with three replicate tanks per concentration. MPs were dispersed ultrasonically before addition to the water. Intestinal samples were collected on 7, 14, and 21 days for the analysis of immune response (tumor necrosis factor-alpha, TNF-α; interleukin-1 beta, IL-1β; interleukin-6, IL-6; interleukin-8, IL-8) and antioxidant activity (superoxide dismutase, SOD; catalase, CAT). Histopathological analysis revealed intestinal wall thinning, villus damage, and epithelial cell detachment in *zebrafish* exposed to both undegraded and degraded MP extracts; however, undegraded MPs induced more severe intestinal damage. Results indicated dynamic changes in cytokine expression: TNF-α decreased initially before increasing, while IL-1β and IL-8 first rose then declined. IL-6 peaked on day 7, dropped by day 14, and increased again on day 21. CAT expression decreased, whereas SOD increased only in the pre-degradation group. Microbial degradation reduced intestinal damage severity, with effects intensifying at higher MP exposure levels. These findings demonstrate that MPs can impair *zebrafish* digestive systems, but microbial degradation mitigates their toxicity. This study underscores the importance of biodegradation as a potential environmental remediation strategy and provides experimental evidence on MPs’ impact on aquatic organisms.

## Simple Summary

Plastic pollution poses an increasing threat to marine life and human health, particularly in accumulation zones such as mangrove wetlands. This study examined the effects of microplastics (MPs) collected from mangrove sediments in Zhanjiang, China, on zebrafish intestinal health. We compared the impacts of untreated MPs with those degraded by microorganisms. Zebrafish were exposed to both types of MPs for 21 days. Results indicated that fish exposed to untreated MPs exhibited more severe intestinal damage and inflammation compared to those exposed to degraded particles, with damage intensifying at higher concentrations and longer exposure durations. These findings suggest that microbial degradation can mitigate the harmful effects of MPs on aquatic organisms, supporting the development of safer bioremediation strategies to address plastic pollution and protect marine ecosystems.

## 1. Introduction

Plastics have become one of the most widespread and persistent pollutants in marine environments, from the poles to the equator [1]. Plastic particles smaller than 5 mm in diameter, found in oceans or on beaches, are classified as microplastics (MPs) [2]. MPs are characterized by their small size, slow degradation rate, and capacity for long-range transport via natural forces such as rivers and ocean currents [3]. Numerous studies have confirmed the global distribution of MPs in oceans, rivers, and sediments [4,5,6]. Sometimes referred to as the “PM2.5 of the ocean,” MPs are readily ingested by marine organisms due to their small size [7].

While larger MPs are generally not absorbed through the intestine wall, their prolonged retention in the digestive tract can cause localized inflammation and disrupt the gut microbiota [8,9]. However, MPs with small particle sizes mainly accumulate in the intestine and are further transferred to the blood. MP exposure can impair growth, development, and reproduction, and can also induce oxidative stress and immune responses [10]. The primary route of exposure is the ingestion of contaminated food and water [11,12]. Therefore, the effects of MP exposure on animal intestines deserve attention. *Zebrafish* are an ideal model for studying intestinal inflammation and host–microbiome interactions due to their small size, high fecundity, fully sequenced genome, and physiological similarities to mammals in intestinal and immune function [13]. Several studies have confirmed that MP exposure over a period of time can induce different levels of inflammation and oxidative stress in the gut of *zebrafish* [8,14].

The extensive root systems of mangrove plants not only mitigate wave energy but also trap pollutants [15]. Consequently, mangrove sediments are considered major sinks for contaminants [16]. Plastics constitute up to 70% of pollutants found in mangrove areas [17]. The abundance of MPs in mangroves is higher than in non-mangrove areas, and MPs are trapped in every layer of sediment [18]. Furthermore, various studies have shown that microorganisms isolated from mangrove sediments can degrade MPs [19,20,21]. Mostly, research on the risks of MP exposure focuses on standard single plastics in the laboratory, which differ from the MPs found in the natural environment. Presently, there are few reports on the effects of extracted MP particles from sediments on the gut of *zebrafish* before and after degradation.

In this study, MPs were extracted from mangrove sediments in Mazhang District, Zhanjiang, and divided into two portions: one subjected to microbial degradation for 120 days, and the other kept undegraded. *Zebrafish* were exposed to various concentrations of degraded MP products or undegraded MPs. Intestinal tissues were collected on days 7, 14, and 21 to compare the effects of degraded and undegraded MPs on intestinal histopathology. We measured the relative expression levels of SOD, CAT, TNF-α, IL-1β, IL-6, and IL-8 mRNA in different MP concentration groups. Our findings provide a scientific basis for bioremediation strategies aimed at mitigating the intestinal toxicity of environmentally sourced MPs.

## 2. Materials and Methods

### 2.1. Extraction and Pretreatment of Microplastics from Mangrove Sediments

Sediment samples were collected from mangrove forests in Mazhang District, Zhanjiang City, Guangdong Province, China. MPs were extracted from the sediments using a wet oxidation method, which included drying, flotation, digestion, and filtration [22]. Polyethylene (PE) powder, identified as the predominant type of MPs in the samples, was spread on a sterile Petri dish and sterilized under ultraviolet light for 6 h with the dish rotated every 30 min. The sterilized PE plastic was then inoculated onto Luria–Bertani (LB) solid medium—a nutrient-rich agar used for bacterial cultivation—and incubated at 30 °C for 3 days. The absence of microbial growth around the powder confirmed sterility.

### 2.2. Zebrafish Acclimation and Rearing Conditions

A total of 2500 healthy adult *zebrafish*, with an average body length of 3.0 ± 0.5 cm and wet weight of 0.34 ± 0.05 g, were purchased from the Zhanjiang Flower and Bird Market. They were identified as *zebrafish* by the Species Identification Group of the Fisheries College of Guangdong Ocean University. The *zebrafish* were subjected to 30 days of adaptive feeding in a 250 L fish tank with an environmental density of 10 tails/L. A circulating water pump was installed to ensure a sufficient oxygen supply, and the water temperature was maintained at 25–28 °C. The light duration was set to 12 h, and the light avoidance duration was set to 12 h. The water was filtered through reverse osmosis, maintaining a pH range of 7.4–7.6 and a conductivity of 500–550 uS/cm. The *zebrafish* were fed twice a day with a commercial pellet feed (Biofish-F100, Shanghai Feixi Biotechnology Co., Ltd., Shanghai, China). According to the manufacturer, the proximate composition of the diet was as follows: crude protein ≥ 48%, crude fat ≥ 10%, crude fiber ≤ 5%, crude ash ≤ 15%, moisture ≤ 12%, phosphorus ≥ 1.0%, lysine ≥ 2.2%. The feeding ration was 3% body weight per feeding with a 12 h interval between feedings. The same diet and feeding regime were maintained throughout the subsequent MP exposure experiment. *Zebrafish* mortality was monitored daily, with immediate removal of deceased individuals to prevent decomposition. Mortality was monitored but not statistically analyzed, as it was not a primary focus of this study.

### 2.3. Microbial Degradation of MPs

Extracted MP particles degradation group: Based on the previous research of our laboratory, K1-1 *Acinetobacter venetianus*—a bacterial strain originally isolated from mangrove sediments—was screened out for degradation experiments due to its significant efficacy in MP degradation. In a 40 mL wide-mouth conical bottle, 4 mL of bacterial solution and 36 mL of MM30 liquid medium were cultured with extracted MP particles. In triplicate, 20, 100, and 500 mg of MPs were added to each bottle and cultured for 120 d in a 180 r/min incubator at 30 °C and were maintained at 40 mL by adding MM30 every 10 days.

The bacterial strain *Acinetobacter venetianus* K1-1, previously isolated from mangrove sediments for its MP-degrading capability, was used for degradation experiments. In 40 mL wide-mouth conical bottles, 4 mL of bacterial suspension and 36 mL of MM30 liquid medium were mixed with extracted MP particles. Triplicate bottles containing 0 (control), 20, 100, or 500 mg of MPs were incubated at 30 °C with shaking at 180 rpm for 120 days. The volume was maintained at 40 mL by adding fresh MM30 medium every 10 days. For the non-degraded MP group, the same amounts of MPs were added to sterile MM30 medium without bacteria and incubated under identical conditions.

### 2.4. Zebrafish Exposure Experiment

*Zebrafish* were randomly distributed into seven experimental groups, each maintained in triplicate tanks (*n* = 3 tanks per group). The groups consisted of the following: a single blank control group (0 mg/L MPs), shared for both degradation conditions; three groups exposed to untreated, pre-degradation MPs at concentrations of 2, 10, and 50 mg/L; and three groups exposed to microbially degraded MPs at the same concentrations of 2, 10, and 50 mg/L.

Each tank consisted of 100 *zebrafish* in 10 L of fully aerated tap water. To ensure homogeneous exposure, the MPs were dispersed using an ultrasonic bath (10 s pulse, 5 s pause, 40 cycles) before being added to the water. The entire volume of water in each tank was replaced every 48 h, and the respective MPs were re-added at that time to maintain the target exposure concentrations throughout the 21-day experimental period. All holding conditions—including water temperature (25–28 °C), pH (7.4–7.6), conductivity (500–550 μS/cm), photoperiod (12 h:12 h light–dark), stocking density (10 fish/L), and feeding regimen (twice daily with commercial pellets at 3% body weight)—were identical to those described for the acclimation period (Section 2.2) to ensure that any observed effects were attributable solely to MP exposure and not to environmental variability.

### 2.5. Sample Collection

Sampling was conducted at 7, 14, and 21 days of exposure. At each time point, 10 *zebrafish* were randomly sampled from each of the three replicate tanks per treatment group, resulting in a total of 30 intestinal samples per group per time point. Intestinal tissues were dissected on a sterile bench, rinsed with phosphate-buffered saline (PBS), and processed for subsequent analysis.

For the 7- and 14-day time points, all 30 samples per group were immediately frozen and stored at −80 °C for subsequent gene expression analysis. For the 21-day time point, the sampling was subdivided: 2 fish from each tank (totaling 6 per treatment group) were fixed in 4% paraformaldehyde for histopathological sectioning, while the remaining 8 fish from each tank (totaling 24 per treatment group) were stored at −80 °C for biochemical and molecular analyses. All samples were processed within 6 months of collection. All animal procedures were approved by the Animal Ethics Committee of Guangdong Ocean University and were conducted in accordance with the relevant guidelines to minimize suffering.

### 2.6. Histological Analysis

Intestinal samples fixed in 4% paraformaldehyde were dehydrated, embedded in paraffin, sectioned at 4 μm thickness, and stained with hematoxylin and eosin (HE). A total of 42 fish (6 per group across 7 groups) were analyzed. For each fish, one representative slide was prepared, and two non-overlapping fields per section were examined under a light microscope (Nikon ECLIPSE E200; Nikon Corporation, Tokyo, Japan) at 200× magnification. Normal intestinal morphology was defined by intact villi, continuous epithelium, visible goblet cells, and absence of necrosis or inflammation. Abnormalities included villus shortening, epithelial detachment, wall thinning, inflammatory infiltration, and cellular necrosis.

### 2.7. Relative mRNA Expression in Intestinal Tissues of Zebrafish

Total RNA was extracted individually from each intestinal sample (30 individuals per group for the 7- and 14-day time points; 24 individuals per group for the 21-day time point) without pooling, using a total RNA extraction kit (TRIZOL Reagent) purchased from Invitrogen, and the concentration of the extracted RNA samples was detected using a nucleic acid protein analyzer. Samples with OD260/280 values of 1.8~2.1 were selected, and the extracted 2 μL RNA was reverse-transcribed into cDNA using a reverse transcription kit. The qPCR reaction system: Green Taq Mix 10 μL of upper and downstream primers 0.4 μL of cDNA 2 μL of ddH2O supplementation 20 μL. The qPCR amplification procedure was pre-denatured at 94 °C for 30 s. The samples were denatured at 94 °C for 5 s, annealed at 55 °C for 15 s, and extended at 72 °C for 10 s for a total of 40 cycles. To determine the changes in the relative expression of target genes, the results were evaluated using the 2^−ΔΔCT^ method, with β-actin as the reference gene. Each sample was analyzed in triplicate. The primers used are listed in Table 1.

### 2.8. Statistical Analysis

Statistical analyses were conducted using SPSS Statistics 27.0. For each treatment group and time point, data from individual *zebrafish* within the same tank were averaged to obtain a single value per tank, as the tank was considered the independent experimental unit. Differences among treatment groups, including the control, undegraded MP groups (2, 10, and 50 mg/L), and degraded MP groups at the same concentrations—were analyzed using one-way ANOVA at each exposure time point (7, 14, and 21 days). A significance threshold of *p* < 0.05 was applied. Results are expressed as mean ± standard error of the tank means (*n* = 3).

## 3. Results

### 3.1. Effects of Microbial Degradation Products of Extracted MP Particles on Intestinal Tract of Zebrafish

**Figure 1:**
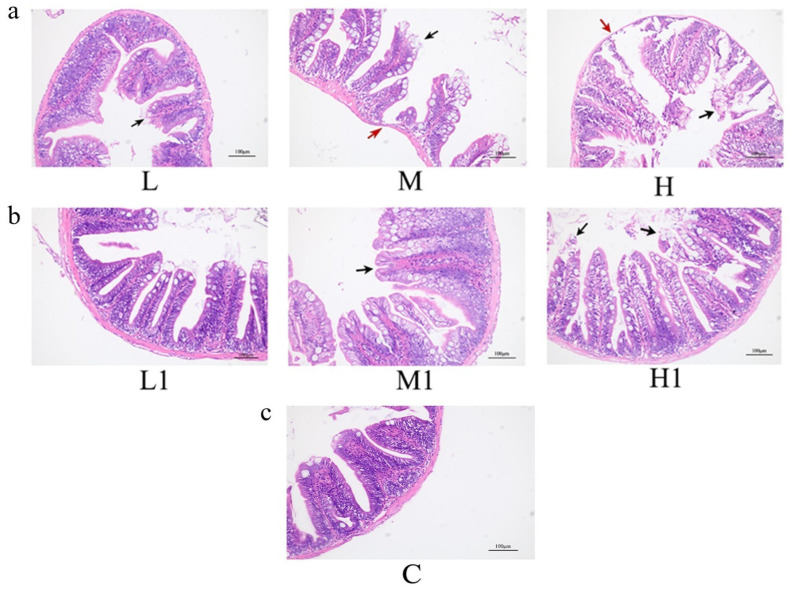
Histological changes in the gut of *zebrafish* after 21 days of exposure. (**a**) MP group before degradation; (**b**) MP group after degradation; (**c**) blank control (0 mg/L); L.L1. Low dose group; M.M1. Medium dose group; H.H1. High dose group; HE, 200×; red arrows indicate thinning of the intestinal wall, while black arrows indicate cell detachment.

After 21 days of exposure, the histological analysis of intestinal tissues was performed (Figure 1). In the control group (0 mg/L), applicable to both degradation conditions, intestinal morphology remained intact, with no noticeable pathological changes throughout the experiment. In the low-dose groups (2 mg/L), both pre- and post-degradation treatments caused only minor villus damage. In contrast, significant alterations were observed in the medium-dose group (10 mg/L). Both treatments exhibited epithelial shedding at villus tips; however, the undegraded MP group displayed markedly thinner intestinal walls, severe villus destruction, and greater cell loss and necrosis. In the high-dose group (50 mg/L), lesions were most pronounced: villus height in the mid-intestine decreased, mucosal epithelium became hypertrophic, and the villous epithelial boundary in the large intestine appeared blurred or absent. Columnar epithelial cells were necrotic and detached, and lymphocytes and goblet cells were scattered. Similar lesions occurred in the degraded MP group, but the intestinal wall was generally more preserved compared to the undegraded group.

### 3.2. Relative mRNA Expression of Oxidative Stress-Related Genes

**Figure 2:**
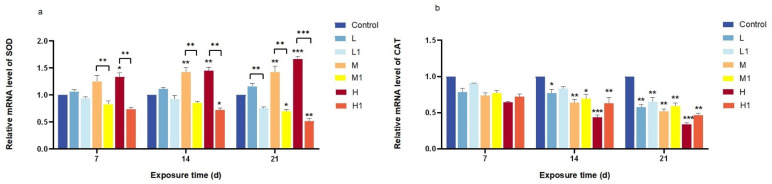
Relative mRNA expression of oxidative stress markers in the intestines of *zebrafish* exposed to extracted MP particles before and after microbial degradation. (**a**) SOD; (**b**) CAT. MPs after degradation are marked with 1 in the lower corner. Data are presented as mean ± SEM; * *p* < 0.05, ** *p* < 0.01, *** *p* < 0.001.

The relative mRNA expression of SOD and CAT in *zebrafish* intestines was analyzed after 7, 14, and 21 days of exposure using qRT-PCR (Figure 2a). In the undegraded MP group, SOD expression increased progressively with exposure duration, while microbial degradation attenuated this effect. Significant differences were detected at medium and high concentrations between the two treatments (*p* < 0.01, *p* < 0.001). After 21 days, SOD expression remained significantly elevated in the undegraded MP group but continued to decline in the degraded MP group, even at low doses (*p* < 0.001). Conversely, CAT expression decreased in all treated groups compared with the control at all time points (Figure 2b). Although CAT levels were consistently lower in the undegraded MP group than in the degraded MP group, the difference between treatments was not statistically significant.

### 3.3. Relative mRNA Expression of Inflammatory Response-Related Genes

**Figure 3:**
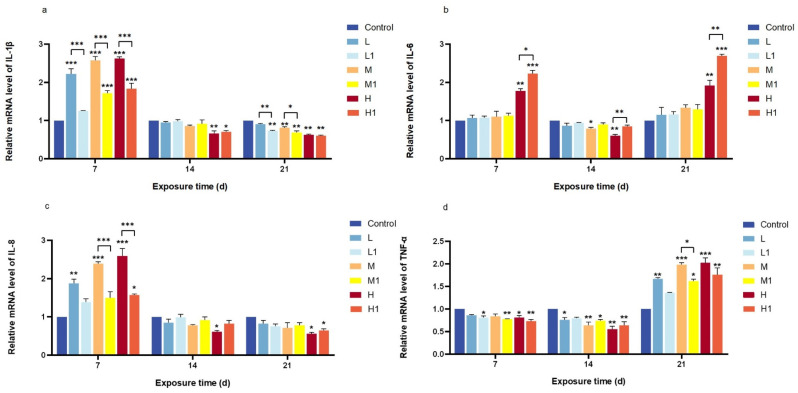
Changes in the relative expression of cytokine mRNA in *zebrafish* before and after degradation of extracted MP particles. (**a**) IL-1β; (**b**) IL-6; (**c**) IL-8; (**d**) TNF-α; MPs after degradation are marked with 1 in the lower corner; * *p* < 0.05, ** *p* < 0.01, *** *p* < 0.001.

IL-1β expression increased significantly in all treatment groups after 7 days, with the undegraded MP group showing the highest levels (*p* < 0.001). However, expression dropped below control levels by days 14 and 21, with a more pronounced decline in the degraded MP group (Figure 3a). IL-6 exhibited a biphasic pattern, increasing at days 7 and 21 but decreasing on day 14, with high-dose effects becoming more evident over time in both treatments (*p* < 0.01) (Figure 3b). IL-8 followed a similar trend to IL-1β, peaking at day 7 before decreasing at later time points; significant differences between undegraded and degraded MPs were only detected at medium and high doses on day 7 (*p* < 0.001) (Figure 3c). TNF-α expression decreased initially but rose again by day 21, with significant differences observed only at the medium dose after 21 days (*p* < 0.05) (Figure 3d).

## 4. Discussion

The *zebrafish* intestine plays a crucial role in digestion, nutrient absorption, waste elimination, and immune defense [23]. Contaminated food is a major source of MP exposure, and the accumulation of MPs in intestinal tissues may lead to local inflammation and affect gut microbes, resulting in various adverse reactions [10,24]. Exposure to polystyrene (PS), a common type of plastic, with a particle size of 5 μm at concentrations of 50 μg/L and 500 μg/L resulted in intestinal damage after 21 d of exposure. This damage includes villus cracking and intestinal cell division [14]. This study showed that exposure of *zebrafish* to MPs can cause intestinal oxidative stress and intestinal inflammation. With an increase in concentration and extension of exposure time, the intestinal inflammation and antioxidant damage in *zebrafish* were more pronounced. However, after microbial degradation, the MP-exposed group had significantly lower intestinal toxicity to *zebrafish* than the MP-exposed group without degradation. Histological analysis revealed intestinal wall thinning, villus damage, and epithelial shedding in both undegraded and degraded MP groups, but lesions were more severe with undegraded MPs. The possible reason for this result is attributed to the fact that MPs are extremely difficult to degrade, and large plastic particles cannot be divided into countless smaller plastic particles in a short period of time [25] and are eventually completely discharged with excreta. However, compared with MPs without degradation, the amount of plastic particles after degradation was relatively reduced. These results demonstrated that the histological sections of different treatment groups showed different degrees of intestinal damage, and the degree of intestinal lesions was not only related to the dose of exposure but also to the degradation of MPs, indicating that microbial degradation of MPs requires further research.

Under external stressors, organisms produce excess reactive oxygen species (ROS), leading to oxidative stress when ROS levels exceed antioxidant capacity [26]. SOD and CAT are the most common metabolic enzymes in the antioxidant stress system of organisms [27]. The main function of CAT is to remove excess hydrogen peroxide from the body and break it down into oxygen and water to protect the cells from hydrogen peroxide damage. SOD is an important metal enzyme, and its main function is to remove excess oxygen free radicals produced in the body, especially superoxide anions (O^2−^). While some studies reported increased SOD and CAT mRNA levels under MP exposure [14], our study found a decrease in CAT expression, with pre-degradation MPs inducing lower CAT levels than post-degradation MPs. However, the relative mRNA expression of the SOD increased only in the group before MP degradation but decreased in the group after degradation, and the reaction became more obvious with the increase in MP exposure dose in both treatment groups. A possible reason for this result is that the MPs used in this study were extracted from the natural environment, and it is difficult to ensure their purity and particle size. For example, the size of MPs determines the extent to which they are transported in the circulatory system, leading to differences in tissue accumulation [28]. Moreover, the extracted MP particles after bacterial degradation, whose components remain to be verified, may be released after bacterial degradation to inhibit the activity of SOD and CAT. As the energy consumed to combat oxidative stress is exhausted, MP exposure leads to a decrease in antioxidant enzymes [29]. Exposure to PS-MPs inhibited CAT activity in *zebrafish* larvae, suggesting that oxidative damage leads to an imbalance in the antioxidant defense system [30]. CAT activity also decreased with increasing MP concentration, suggesting oxidative damage and stress. The composition of the products after plastic biodegradation is complex and may be toxic to the ecosystem. However, there is still a lack of a summary of the environmental behavior of the degradation products. Therefore, it is very necessary to study the composition analysis of MP degradation products for the subsequent work of improving biodegradation.

*Zebrafish*, as aquatic vertebrates, possess a well-developed immune system comprising various tissues and cytokines that contribute to maintaining internal homeostasis. Studies have indicated that cytokine expression levels serve as effective biomarkers of inflammation in *zebrafish* [8]. To further investigate the effects of MP exposure on the intestinal tract of *zebrafish*, this study measured the relative mRNA expression levels of four immune-related cytokines: IL-1β, TNF-α, IL-6, and IL-8. IL-6 is a pivotal cytokine implicated in the development of chronic inflammation [31]. In this study, it was found that the mRNA expression level of IL-6 in both groups before and after MP degradation displayed an initial increase, followed by a decrease and then an increase. However, only the high-dose group showed statistically significant differences as compared to others. These findings imply that with an increase in exposure time, MPs can enter the interior of cells, interact with intracellular proteins, and affect IL-6 production by regulating cell-signaling pathways [32]. TNF-α, secreted primarily by monocytes, acts as an important cellular activator that regulates tissue metabolism and stimulates the synthesis and release of other cytokines [33]. Its principal functions include inhibiting and killing tumor cells, combating infections, and promoting inflammatory responses. In this study, the mRNA expression of TNF-α in *zebrafish* initially decreased and then increased relative to the control group. Significant differences from the control were observed at low, medium, and high MP concentrations after 7 and 14 days of exposure; however, there was no significant difference observed before and after the degradation of MPs. Notably, a significant difference was observed only after 21 d of exposure. Exposure to 0.5 μm polystyrene microplastics significantly increased the mRNA level of TNF-α in *zebrafish* viscera compared to the control group [8], a result consistent with the findings in the 21-day exposed group of this study, which may be related to the abnormal activation of the immune system. This suggests that MP pollution may negatively affect the immune system of *zebrafish*, impairing their immune function and leading to chronic inflammation and disease. IL-1β is an important pro-inflammatory cytokine produced by activated macrophages in response to exogenous stimuli [34], which can regulate the immune response, and cell metabolism and induce the gradual release of other pro-inflammatory factors. MP exposure significantly increased the expression and release of IL-1β in *zebrafish* [35]. This may be because MPs activated the immune system after entering the *zebrafish*, leading to the increased secretion of interleukin IL-1β. In contrast, our results demonstrated an increase in IL-1β levels only after 7 days of MP exposure, with a decline observed after 14 and 21 days. IL-8 is a chemokine secreted by mononuclear macrophages and can induce neutrophils to release active products that participate in inflammatory responses [36]. This study found that the relative expression of IL-8 mRNA in response to MP exposure initially increased and then decreased compared to the control group, with no significant differences among exposure groups. These results suggest that MPs may reduce the resistance of white blood cells to pathogenic bacteria, leading to diminished IL-8 expression. This suggests that MP contamination may affect the immune system of *zebrafish*, making them more vulnerable to infections and diseases.

In this study, both degraded and non-degraded MP groups caused immune and oxidative stress damage in *zebrafish*. However, the relative mRNA expression levels of TNF-α, IL-1β, and IL-8 decreased after degradation compared to the non-degraded group. This may be attributed to the reduced particle size of MPs following bacterial degradation, facilitating their excretion in feces, or to the diminished toxicity of certain harmful substances, such as associated additives, thereby reducing the overall harm to *zebrafish*. Previous studies have indicated that MPs of smaller particle sizes can be eliminated via fecal excretion after ingestion by fish [37], resulting in reduced toxicity—a finding consistent with the results of this experiment. The effects of MPs on IL-1β, IL-6, IL-8, TNF-α, SOD, and CAT have not been fully studied. Moreover, the influence of MPs varying in type, shape, and concentration on these biomarkers may differ significantly, and their impacts in real-world environments remain to be elucidated. Therefore, further research is necessary to examine the toxic effects of MPs under varying conditions such as particle size, polymer type, and exposure concentration.

## 5. Conclusions

This study demonstrated that the intestinal toxicity of MPs extracted from mangrove sediments decreased significantly after microbial degradation compared with undegraded MPs. The severity of toxicity was influenced by both concentration and exposure duration, with higher doses and longer exposure times causing more pronounced intestinal inflammation and oxidative stress. Although microbial degradation mitigated these adverse effects, toxicity was not completely eliminated. These findings offer important insights into the potential of microbial degradation as a strategy to reduce MP-induced toxicity in aquatic organisms and provide a foundation for future bioremediation research.

## Figures and Tables

**Table 1:** Primer sequences for real-time PCR.

Gene	Forward (5′–3′)	Reverse (3′–5′)
IL-1β	GATTCGCAGATGGTGGAGATGGAC	TCGTCTTTGGATGGAAGCACAGC
IL-6	TCAACTTCTCCAGCGTGATG	TCTTTCCCTCTTTTCCTCCTG
IL-8	AACATGGAGGTCATTGCCACTGTG	GAGGTAGAATTTGGAGGGAGGGTA
TNF-α	GTCGGGTGTATGGAGGGTGTTTG	CTGGTCTTATGGAGCGTGAAGC
SOD	GTCCGCACTTCAACCCTCA	TCCTCATTGCCACCCTTCC
CAT	AGGGCAACTGGGATCTTACA	TTTATGGGACCAGACCTTGG
β-actin	GTGATGGACTCTGGTGATGGTGTG	CACGCTCGGTCAGGATCTTCATC

## Data Availability

The data presented in this study are available on request from the corresponding author. The data are not publicly available due to privacy protection.

## Abbreviations

MPs	Microplastics
SOD	Superoxide dismutase
CAT	Catalase
HE	Hematoxylin and eosin
PE	polyethylene
LB	Luria–Bertani
TNF-α	tumor necrosis factor-alpha
IL-1β	interleukin-1 beta
IL-6	interleukin-6
IL-8	interleukin-8
PBS	phosphate-buffered saline
PS	polystyrene
ROS	reactive oxygen species
